# Association of putatively adaptive genetic variation with climatic variables differs between a parasite and its host

**DOI:** 10.1111/eva.13234

**Published:** 2021-04-08

**Authors:** Sheree J. Walters, Todd P. Robinson, Margaret Byrne, Grant W. Wardell‐Johnson, Paul Nevill

**Affiliations:** ^1^ ARC Centre for Mine Site Restoration School of Molecular and Life Sciences Curtin University Bentley WA Australia; ^2^ School of Earth and Planetary Science Curtin University Bentley WA Australia; ^3^ Biodiversity and Conservation Science Department of Biodiversity, Conservation and Attractions Bentley WA Australia; ^4^ School of Molecular and Life Sciences Curtin University Bentley WA Australia; ^5^ Trace and Environmental DNA Laboratory School of Molecular and Life Sciences Curtin University Bentley WA Australia

**Keywords:** comparative genomics, genome scans, genotype–environment association, landscape genetics, loranthaceae, mistletoe, parasitic species

## Abstract

Parasitism is a pervasive phenomenon in nature with the relationship between species driving evolution in both parasite and host. Due to their host‐dependent lifestyle, parasites may adapt to the abiotic environment in ways that differ from their hosts or from free‐living relatives; yet rarely has this been assessed. Here, we test two competing hypotheses related to whether putatively adaptive genetic variation in a specialist mistletoe associates with the same, or different, climatic variables as its host species. We sampled 11 populations of the specialist mistletoe *Amyema gibberula* var. *tatei* (*n* = 154) and 10 populations of its associated host *Hakea recurva* subsp. *recurva* (*n* = 160). Reduced‐representation sequencing was used to obtain genome‐wide markers and putatively adaptive variation detected using genome scan methods. Climate associations were identified using generalized dissimilarity modelling, and these were mapped geographically to visualize the spatial patterns of genetic composition. Our results supported the hypothesis of parasites and host species responding differently to climatic variables. Temperature was relatively more important in predicting allelic turnover in the specialist mistletoe while precipitation was more important for the host. This suggests that parasitic plants and host species may respond differently to selective pressures, potentially as a result of differing nutrient acquisition strategies. Specifically, mistletoes acquire water from hosts (rather than the abiotic environment), which may provide a buffer to precipitation as a selective pressure. This work deepens and complements the physiological and other ecological studies of adaptation and provides a window into the evolutionary processes that underlie previously observed phenomena. Applying these methods to a comparative study in a host–parasite system has also highlighted factors that affect the study of selection pressure on nonmodel organisms, such as differing adaptation rates and lack of reference genomes.

## 1 INTRODUCTION

Parasitism is ubiquitous across the tree of life (Musselman & Press, 1995; Poulin, 2011; Poulin & Morand, 2000) with host–parasite interactions driving evolution in both partners. These interactions are typically investigated with respect to coevolution (e.g. Laine, 2008; Lopez Pascua et al., 2012), and due to their host‐dependent lifestyle, parasites may adapt to the abiotic environment in similar ways to their hosts as they have the same spatial distribution and thus experience the same temperature and precipitation regimes (e.g. Gorter et al., 2016). Alternatively, the host‐dependent lifestyle may provide a buffer between a parasite and their abiotic environment, resulting in different adaptive responses compared to their hosts. These opposing alternatives make host–parasite systems interesting models for studying local adaptation (Kaltz & Shykoff, 1998; Kawecki & Ebert, 2004).

Previous studies on host–parasite systems have shown that local adaptation can be influenced by heterogeneity of the abiotic environment (Nuismer & Gandon, 2008; Thompson, 2005; Wolinska & King, 2009), with evidence for the influence of temperature and nutrient levels emerging across plant pathogens (Laine, 2007, 2008; Mboup et al., 2012) and other parasitic microorganisms (Gorter et al., 2016; Lopez Pascua et al., 2012; Mitchell et al., 2005). An increasing number of local adaptation studies integrate the abiotic environment within laboratory experiments. However, few have examined the association of adaptation with climatic gradients in field‐based settings. Such studies could provide crucial understanding of adaptation patterns in natural populations (Thompson, 2005). Furthermore, while adaptation studies across a range of taxonomic groups have recently benefitted from the analysis of genome‐wide markers (reviewed in Ahrens et al., 2018), these approaches have rarely been applied to host–parasite systems (but see Hartmann et al., 2019; Walters et al., 2020 for recent examples).

Mistletoes are aerial parasites that acquire water and mineral nutrients via a specialized haustorium structure, unique to parasitic plants (Kuijt, 1969; Musselman & Press, 1995). Mistletoes are important for the functioning of ecosystems worldwide (Press & Graves, 1995; Press & Phoenix, 2005; Watson, 2001), providing food resources for fauna (Press & Phoenix, 2005) and increasing nutrient cycling within plant communities (March & Watson, 2010). Mistletoe–host systems provide a useful model for local adaptation studies as the parasite is sessile, vector‐dispersed and entirely reliant on a single individual host for survival and persistence (Calder, 1983; Press & Graves, 1995). Climatic factors are known to influence the distribution, survival (e.g. de Buen & Ornelas, 2002; Scalon & Wright, 2015) and genetic variation in mistletoes (e.g. Ramírez‐Barahona et al., 2017). For example, Ramírez‐Barahona et al. (2017) found that patterns of genetic variation in a mistletoe varied along precipitation and seasonality gradients, which are also important drivers of local adaptation in autotrophic plants (Shryock et al., 2017; Steane et al., 2017). However, few studies have examined whether signatures of selection in parasitic plants match those of their hosts.

A parasitic lifestyle may enable mistletoes to adapt to climatic gradients in different ways from their host species. Mistletoes are susceptible to xylem cavitation when water potentials drop too low (Ehleringer & Marshall, 1995), and they must have higher transpiration rates than hosts to maintain a positive water gradient (Ehleringer et al., 1985; Stewart & Press, 1990). Therefore, climatic factors that affect transpiration rates (i.e. temperature) may provide a stronger selection pressure for mistletoes than that experienced by their hosts. Mistletoes also have their own ecological or climatic niche requirements and will only occur where suitable abiotic conditions overlap with appropriate host species (e.g. Lira‐Noriega & Peterson, 2014; Ramírez‐Barahona et al., 2017).

A recent study by Walters et al. (2020) found associations between genome‐wide variation and climatic variables to be different between a parasitic plant and a sympatric autotrophic species. However, the study species (*Nuytsia floribunda*) was a generalist parasite that has numerous host species (Calladine et al., 2000), compared to specialist parasites that utilize a single, or few, often related, host species. Due to their greater host specialization, patterns of genome‐wide variation in specialist parasites may be more similar to patterns of their host, compared to generalist parasites that may have patterns of variation not associated with that of their hosts due to environmental buffering afforded by multiple host relationships.

We sought to test two competing hypotheses in our study of a specialist mistletoe and its specific host species. Our null hypothesis was that putatively adaptive genetic variation in the mistletoe would associate with the same climatic variables as its host species. Due to the semi‐arid climate of the study landscape, precipitation may be relatively more important than temperature in predicting allelic turnover for both species. Our alternative hypothesis was that putatively adaptive variation in the specialist mistletoe would associate with different climatic variables than its host species, due to environmental buffering provided by the host species. Specifically, as mistletoes rely on higher transpiration rates to create a positive water gradient with the host (Ehleringer et al., 1985; Stewart & Press, 1990), temperature may be relatively more important than precipitation in predicting allelic turnover for the mistletoe.

Here, we applied a genotyping‐by‐sequencing approach to test these hypotheses by examining the patterns of genome‐wide variation between a mistletoe *Amyema gibberula* var. *tatei* (Blakely) Barlow (family Loranthaceae) and its host species, *Hakea recurva* Meisn. subsp. *recurva* (family Proteaceae). We aimed to develop our understanding of the association of putatively adaptive genetic variation along climatic gradients between parasites and their associated host species. Specifically, we examined (a) the association of putatively adaptive variation with climatic variables and the relative importance of temperature, precipitation and geographic distance with allelic turnover and (b) the predicted spatial pattern of putatively adaptive variation.

## 2 MATERIALS AND METHODS

### 2.1 Study species and sample collection

**FIGURE 1:**
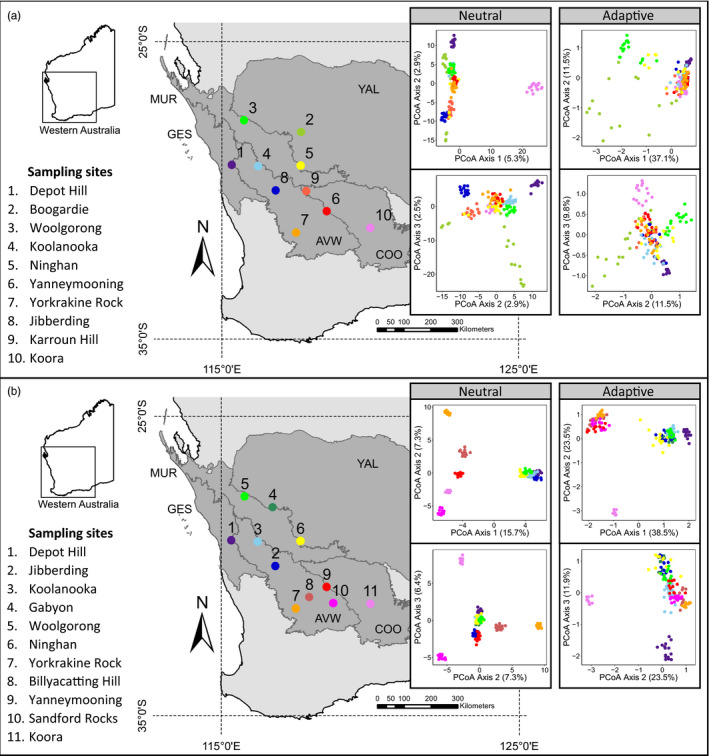
Sampling sites and principal coordinate analysis (PCoA) of putatively neutral and putatively adaptive genetic variation for (a) *Hakea recurva* subsp. *recurva* and (b) *Amyema gibberula* var. *tatei*. Neutral data sets (14,848 and 1631 SNPs for each species, respectively) contained putatively neutral SNPs not identified as outliers, or with significant environment association, in any genome scan method. Adaptive data sets (35 and 36 SNPs for each species, respectively) contained putatively adaptive SNPs identified by two or more genome scan methods. Maps show the geographic location of sample sites across five bioregions (Avon Wheatbelt, AVW; Coolgardie, COO; Geraldton Sandplains, GES; Murchison, MUR; Yalgoo, YAL). Samples within the PCoAs are colour‐coded by site, and the percentage on each axis indicates how much genome‐wide variation between individuals was explained by the axis

The specialist mistletoe *Amyema gibberula* var. *tatei* and its host *Hakea recurva* subsp. *recurva* have a widespread distribution in south‐western Australia, spanning *c*. 500 km north‐south and *c*. 300 km east‐west (Figure 1). Temperature and precipitation vary across the species’ distribution with a semi‐arid climate in the north‐east and dry Mediterranean climate in the south‐west. The host occurs as a tree or shrub to 6 m in height, is pollinated by insects and has gravity/wind dispersed seed. In contrast, the mistletoe is a hemiparasitic aerial shrub that occurs only on *Hakea* species and almost entirely on *H*. *recurva subsp*. *recurva* (Start, 2015). Like other *Amyema* species in Australia, flowers are bird‐pollinated and the fleshy fruit is dispersed by the mistletoe bird, *Dicaeum hirundinaceum* (Liddy, 1983).

Sampling locations were distributed throughout the entire range of the mistletoe to capture the full geographic space, as well as the spatial envelope of precipitation and temperature that the species occupies. To allow comparison between species, host populations were sampled to cover a similar geographic and climatic range. Host populations were generally found on granite outcrops, rocky ridges and rocky sandstone slopes while the distribution of mistletoe populations was dependent on the availability of host plants. Leaf tissue was collected from 160 host and 154 mistletoe plants with 10 – 16 individuals per species sequenced from 10 and 11 populations, respectively (Tables S1 and S2). Only one mistletoe was collected from each host plant, and we aimed for a minimum sampling distance of 20 m between individuals to avoid sampling related individuals (achieved for 93% of host and 85% of mistletoe individuals). Host plants and attached mistletoes were sampled together when possible, with approximately 61% of host individuals collected with a respective mistletoe. Samples were stored on silica gel, and the location of each individual sampled was recorded using a GARMIN eTrex® 10 GPS.

### 2.2 Climatic data assemblage

Climatic data for fifteen variables covering annual and quarterly temperature averages, annual and quarterly precipitation totals, and seasonality, were downloaded in raster format at 1 km cell resolution from the Worldclim2.0 database (Fick & Hijmans, 2017; Hijmans et al., 2005). Point information was extracted using the coordinates of each sampled individual using the Spatial Analyst toolbox in ArcMap (ESRI, 2019). Variables were split into temperature and precipitation groups, and Spearman rank correlation tests were performed using the *stats* package in R (R Core Team, 2019) to assess pairwise collinearity between variables within each group. To minimize inclusion of highly correlated factors (Rellstab et al., 2015), we selected variables that had within‐group pairwise correlations of |*r*| < 0.8 and that varied across the study area. This data set comprised four temperature variables (isothermality, temperature seasonality, mean temperature of the wettest quarter and mean temperature of the driest quarter) and three precipitation variables (annual precipitation, precipitation seasonality and precipitation of the warmest quarter). Variables in the final data set were plotted in the R package *raster* v3.0‐12 (Figure S1; Hijmans, 2020), and population‐level means are provided in Tables S1 and S2.

### 2.3 SNP generation and bioinformatics

DNA for each species was extracted from *c*. 40 mg of silica‐dried leaf tissue using the CTAB method of Doyle and Doyle (1990) with the addition of 2% PVP (polyvinylpyrrolidone) and 0.2% β‐mercaptoethanol to the extraction buffer. DNA was visualized on a 1% agarose gel and quantified using a Qubit 2.0. Samples with DNA concentration above 80 ng/µl were diluted to 70 ng/µl, and approximately 20 µl of purified DNA in TE buffer was sent for individual genotyping at Diversity Arrays Technology Pty Ltd. DNA from a subset of individuals (4.4% for the host and 14.3% for the mistletoe) was replicated across multiple plates, but processed independently, to ensure between‐plate genotyping consistency.

Samples were genotyped using DArTseq, which combines double digest complexity reduction methods and next‐generation sequencing to assay millions of markers for genome‐wide single nucleotide polymorphisms (SNPs; Kilian et al., 2012; Sansaloni et al., 2011). Genome reduction was undertaken using a combination of two methylation‐sensitive restriction enzymes, *Pst*I/*Mse*l for the host and *Pst*I/*Hpa*II for the mistletoe, with the digestion and adaptor ligation process described by Kilian et al. (2012). High‐density sequencing was run on the Illumina HiSeq 2500 platform and sequence alignment performed *de novo* using Diversity Array Technology's propriety analytical pipeline as prior genomic information was not available for either species (or related species). Sequences were filtered to remove those with a Phred score <30, and the remainder were collapsed into identical sequences. SNP marker calling was performed with Diversity Array Technology's propriety DArTsoft14 pipeline. Approximately 25% of samples were regenotyped as technical replicates to allow a measure of DArTseq reproducibility at each locus to be calculated. NCBI BLAST (Camacho et al., 2009) of bacteria and fungi databases was used to remove microbial DNA from barcoded sequences. Remaining sequences were trimmed and split into individual organism data.

Further quality control filtering was performed using the package *dartR* v1.1.11 (Gruber et al., 2018) in *R* (R Core Team, 2019) to ensure only high‐quality data were used for downstream analysis. Specifically, we removed replicates of individuals from the SNP data sets and then filtered the data set to retain: (1) SNPs with less than 5% missing data, (2) SNPs with DArTseq reproducibility score >0.98, (3) SNPs with minor allele frequency greater than 5% and (4) individuals with <20% missing data. Downstream genetic analyses typically assume loci are not closely linked (see Hoban et al., 2016). Therefore, as a final filtering step, we randomly selected only one SNP per fragment to be retained in the data set.

### 2.4 Landscape and population genetic analyses

To separate putatively adaptive and putatively neutral genetic variation, we used a combination of one population differentiation (PD) test and two environment association (EA) analyses to detect loci under selection. Specifically, we used *pcadapt* v4.1.0 (Luu et al., 2017) in R for PD testing, which uses principal component analysis to identify SNPs with excessive association to population structure, but not to specific environmental variables. In comparison, EA approaches account for neutral population structure to detect SNPs with significant associations to environmental variables (e.g. temperature and precipitation; Hoban et al., 2016), although, unlike PD tests, they can lose discriminatory power under certain demographic scenarios (Forester et al., 2018; Lotterhos & Whitlock, 2015). The two EA approaches used in this study each have a different statistical approach in correcting for population structure: latent factor mixed models (LFMM; Caye et al., 2019; Frichot et al., 2013) use a least‐squares estimation approach and BayPass (Gautier, 2015) uses Bayesian hierarchical modelling.

*pcadapt* uses a robust Mahalanobis test statistic to identify SNPs in which ɀ‐scores do not follow the same distribution as those of the larger data set, and these are considered as outliers (Luu et al., 2017). We set the false discovery rate (FDR) set to 5%, and the optimum number of principal components (PCs) was identified by running *pcadapt* with *K* = 10 and interpreting the scree plot using Cattell's rule (Cattell, 1966). Secondly, *pcadapt* was run with the optimum number of PCs and a MAF threshold of 0.05 to calculate the test statistic and *p*‐values for each locus. For FDR control, *p*‐values were transformed into *q*‐values using the R package *qvalue* v2.18.0 (Storey et al., 2019) and SNPs with *q* < 0.05 were identified as outliers.

LFMM 2.0 uses allele frequency data and an imputed number of latent factors to calculate an exact solution for latent factor regression models, while controlling for confounding variables (Caye et al., 2019). The analysis was implemented in the R package *lfmm* v1.0 (Caye et al., 2020). We estimated the number of latent factors (*K*) following the package vignette, performing principal component analysis (PCA) on the data set using the R function *prcomp* (R Core Team, 2019). Results of the PCA were plotted as screeplots and interpreted using Cattell's rule (Cattell, 1966). Missing genetic data were imputed in the R package *LEA* v2.8 (Frichot & François, 2015) using *K* latent factors, and each climatic variable was scaled to a standard deviation of one. LFMM analysis was run for each climatic variable through ridge estimates using *K* latent factors. For each climatic variable, ɀ‐scores were used to derive a genomic inflation factor (λ) that was used to adjust *p*‐values based on a chi‐squared (χ^2^) distribution (François et al., 2016). For FDR control, a Benjamini–Hochberg *p*‐value correction was applied according to Frichot and François (2015) and SNPs with *q* < 0.05 were considered to have a significant SNP–environment association.

BayPass tests for covariance between population‐level allele frequencies and environmental variables while correcting for demographic effects (Gautier, 2015). The core model in BayPass was run four times with default settings in addition to nval of 100,000, burnin of 50,000, npilot of 30 and pilotlength of 5000, with results averaged over runs. The XtX statistic was calibrated using the *simulate*.*baypass* function according to the manual to create a pseudo‐observed data set that was run in BayPass using the same settings as the core model. The results were used to identify SNPs with an XtX statistic below 3% (representing balancing selection) or above 97% (representing directional selection), which were considered outliers. To identify association with environmental variables, outlier SNPs (both balancing and directional) were removed to create a neutral data set that was run in BayPass with the same settings as the core model. The average of four runs was used to create a neutral covariance matrix. Finally, the auxiliary model was run in BayPass using the neutral covariance matrix and the seven climatic variables with the same settings as the core model. Bayes factors were obtained from the mean of four runs and were transformed into deciban units (dB) using the 10log_10_(BF) transformation. Values of 20 deciban units or more were considered as strong evidence for significant SNP–environment associations (Kass & Raftery, 1995).

We plotted the total number of significant SNPs for each method using the R package *VennDiagram* v1.6.20 (Chen, 2018) and split the SNPs into putatively neutral and putatively adaptive data sets. Putatively neutral SNPs were considered to be those not identified as outliers, or with significant environment association, by any genome scan method, and putatively adaptive SNPs were considered to be those identified by two or more methods (Forester et al., 2018). To minimize the inclusion of false positives in the putatively adaptive data set, we used a consensus approach as recommended by De Mita et al. (2013). We then estimated global and pairwise *F_ST_* (Weir & Cockerham, 1984) between populations for putatively neutral and putatively adaptive data sets using the R package *hierfstat* v0.04‐22 (Goudet, 2005), with the latter used as input for the spatial modelling. Additionally, we used principal coordinate analysis (PCoA) in the R package *dartR* v1.1.11 (Gruber et al., 2018) to examine differences in genetic structure for putatively neutral and putatively adaptive data sets and plotted the first three PCoA axes using R package *ggplot2* v3.2.1 (Wickham, 2016).

### 2.5 Landscape genetic modelling

Generalized dissimilarity modelling (GDM; Ferrier, 2002; Ferrier et al., 2002) was used to examine and compare the association of genome‐wide variation with climatic gradients between the mistletoe and host. Specifically, we compared (i) the association of putatively adaptive and putatively neutral genetic variation with climatic variables, using the method described by Fitzpatrick and Keller (2015) on applying GDMs to genome‐wide markers and (ii) the relative importance of temperature, precipitation and geographic distance in predicting allelic turnover, using variation partitioning (Borcard et al., 1992). Pairwise *F_ST_* matrices (scaled to between zero and one) were used as the biological response variable, and predictor data sets were assembled with geographic coordinates of each population along with the seven climatic variables.

GDM analysis was implemented in the R package *gdm* v1.3.11 (Manion et al., 2018) to assess the relative importance of each climatic variable against allelic turnover. For each data set, we used a backward elimination procedure with 500 permutations and three splines to measure significance (*α* = 0.05) of each climatic variable (Ferrier et al., 2007; Fitzpatrick et al., 2013). Only significant climatic variables were retained in the final GDM models. We summed the spline coefficients to quantify the relative importance of each predictor variable (Fitzpatrick et al., 2013; Yates et al., 2019).

Monotonic I‐spline turnover functions were calculated for predictor variables in the final GDM models, and these were mapped using *ggplot2* to visualize the relationship between allelic turnover and climatic variables. Spline height represented the amount of explained genetic variation, when holding all other variables constant, and spline slope indicated the rate of genetic differentiation across the range of the predictor (Fitzpatrick & Keller, 2015; Fitzpatrick et al., 2013). Next, we partitioned the deviance resulting from the GDM models into geographic distance, temperature and/or precipitation variables to evaluate the contributions of each in explaining allelic turnover (Borcard et al., 1992; Yates et al., 2019). Partitioned deviance was plotted in Venn diagrams using the R package *eulerr* v6.0.0 (Larsson, 2019).

To examine the predicted spatial patterns of putatively adaptive and putatively neutral genetic variation, we visualized the GDM models using the spatial interpolation method of Fitzpatrick and Keller (2015). Briefly, we used fitted GDMs to transform significant climatic variables into genetic importance values and then used PCA to reduce the transformed variables into three PCs, which were composited into an RGB raster image (R = PC1, G = PC2 and B = PC3). Similar colours correspond to similar predicted patterns of genetic composition. To compare mapped genetic patterns between the two data sets (putatively neutral SNPs; putatively adaptive SNPs) for each species, we used Procrustes analysis (Peres‐Neto & Jackson, 2001) to measure and map the similarity of multivariate configuration following the approach of Fitzpatrick and Keller (2015). Procrustes residuals measure the absolute difference in patterns of predicted genetic compositions between putatively neutral and putatively adaptive data sets for each species. Further, to allow direct comparison between species, we scaled residuals by the largest and smallest value observed across both species following the method of Walters et al. (2020). Finally, residuals were mapped geographically to identify areas with the largest differences in genetic composition patterns between SNP data sets.

## 3 RESULTS

### 3.1 SNP generation

DArTseq technologies produced SNP data sets that comprised 118,880 SNPs across 80,296 loci for host *Hakea recurva* subsp. *recurva* (*n* = 160) and 15,187 SNPs across 10,415 loci for specialist mistletoe *Amyema gibberula* var. *tatei* (*n* = 154). All replicates had greater than 97% genetic similarity. Following further quality control filtering, the working data sets comprised 15,422 SNPs for the host and 2055 SNPs for the mistletoe (Walters et al., 2021), with global missing data of 1.20% and 1.12%, respectively. All individuals were retained in the data sets for both species.

### 3.2 Landscape and population genetic analyses

*pcadapt* identified 488 SNPs as outliers for the host (mean *χ* ^2^ = 6.32, *df* = 5) and 225 SNPs as outliers for the mistletoe (mean *χ* ^2^ = 5.79, *df* = 2). LFMM identified 59 significant SNP–environment associations for the host involving 47 SNPs. In contrast, LFMM identified 272 significant SNP–environment associations for the mistletoe involving 135 SNPs. Similarly, BayPass identified 88 SNP–environment associations involving 81 SNPs for the host and 107 SNP–environment associations for the mistletoe involving 105 SNPs. For both EA approaches, the number of significant SNP–environment associations varied between climatic variables and no SNPs for either species were significantly associated across all variables (Table 1).

**TABLE 1:** Number of SNPs with significant environment association for *Hakea recurva* subsp. *recurva* (*n* = 15,422 SNPs) and *Amyema gibberula* var. *tatei* (*n* = 2,055 SNPs). Environment association analyses (LFMM and BayPass) were run on each species across seven climatic variables. The total number of unique SNPs identified by the two approaches is shown for each variable

Climatic variable	*Hakea recurva* subsp. *recurva*			*Amyema gibberula* var. *tatei*		
	LFMM	BayPass	Total	LFMM	BayPass	Total
IT (BIO3)	3	0	3	102	1	103
TS (BIO4)	7	5	12	15	0	15
MTWQ (BIO8)	2	36	38	18	54	66
MTDQ (BIO9)	1	1	2	0	13	13
AP (BIO12)	15	20	27	48	19	60
PS (BIO15)	4	13	15	79	0	79
PWQ (BIO18)	27	13	35	10	20	29

Abbreviations: AP, annual precipitation; IT, isothermality; MTDQ, mean temperature of the driest quarter; MTWQ, mean temperature of the wettest quarter; PS, precipitation seasonality; PWQ, precipitation of the warmest quarter; TS, temperature seasonality.

**FIGURE 2:**
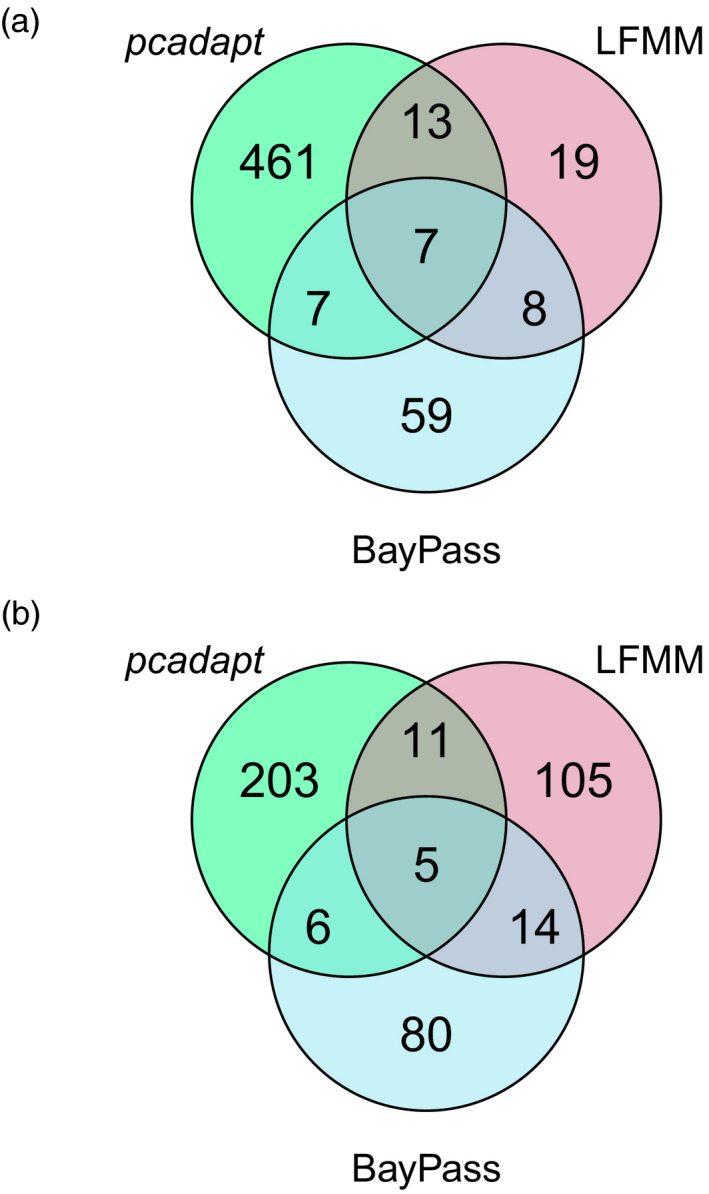
Venn diagrams of SNPs identified by three genome scan methods for (a) *Hakea recurva* subsp. *recurva* and (b) *Amyema gibberula* var. *tatei*. The diagrams show a comparison between SNPs obtained from *pcadapt*, LFMM and BayPass methods. For the environment association methods (LFMM and BayPass), SNPs that were significant for multiple environmental variables were only included once in the Venn diagram

Overall, 574 SNPs were identified by at least one analytical method for the host (Figure 2a) and those same methods identified 424 SNPs for the mistletoe (Figure 2b). To minimize the inclusion of false positives due to biases of individual methods, only SNPs identified by more than one genome scan method were included in putatively adaptive data sets (35 and 36 SNPs for the host and mistletoe, respectively). All these SNPs showed either higher than expected differentiation between populations or significant association with climatic variables in multiple methods, although they may not all be directly affected by selection but could be physically linked to loci under selection. By contrast, putatively neutral data sets (14,848 and 1631 SNPs for the host and mistletoe, respectively) comprised SNPs not identified as outliers, or with significant environment association, in any genome scan method. Overall, global *F_ST_* was greater in the mistletoe than the host. Specifically, *F_ST_* values for the putatively neutral data sets were 0.079 and 0.330 for the host and mistletoe, respectively, while values for the putatively adaptive data sets were 0.370 and 0.734 (host and mistletoe, respectively).

PCoA identified differing genetic structure between species and data sets (Figure 1). For the putatively neutral data sets, the first PCoA axis separated out the most south‐eastern population of the host while the remaining populations were separated along the second and third axes, collectively explaining 10.7% of genetic variation (Figure 1a). By contrast, the first PCoA axis separated the northern and southern populations of mistletoe while the second and third axes distinguished between southern populations, collectively explaining 29.4% of genetic variation (Figure 1b). For the putatively adaptive data sets, the first PCoA axis separated out the north‐eastern populations, with host individuals separated along the second axis, and the first three axes explained 58.4% of the genetic variation (Figure 1a). In the mistletoe, the first PCoA axis separated the northern and southern populations but the second axis separated out the most south‐eastern population, and the first three axes explained 73.9% of genetic variation (Figure 1b).

### 3.3 Landscape genetics modelling

Following the GDM backward elimination procedure, no predictor variables were significant (*α* = 0.05) for the putatively neutral data set of the host, although we opted to use mean temperature of the wettest quarter (*p* = 0.06) in the final GDM model (Table 2). Two significant variables—geographic distance and annual precipitation—were retained in the GDM model of the putatively adaptive data set for the host. Similarly, the only significant predictor variable retained in the GDM model of the putatively neutral data set for the mistletoe was mean temperature of the wettest quarter while three variables—geographic distance, temperature seasonality and mean temperature of the wettest quarter—were retained in the GDM model for the putatively adaptive data set. Overall, predictor variables explained a similar percentage of model deviance for both species (Table 2).

**TABLE 2:** Model fit of generalized dissimilarity modelling for *Hakea recurva* subsp. *recurva* and *Amyema gibberula* var. *tatei* data sets. Putatively neutral data sets contained SNPs that were not identified as outliers, or with significant environment association, in any genome scan method (14,848 and 1,631 SNPs for each species, respectively). Putatively adaptive data sets contained only SNPs identified by two or more genome scan methods (35 and 36 SNPs for each species, respectively). Models contain only significant predictor variables (*p* < 0.05), except for the *H*. *recurva subsp*. *recurva* neutral data set (*p* = 0.06)

Model	*Hakea recurva* subsp. *recurva*		*Amyema gibberula* var. *tatei*	
	Neutral data set	Adaptive data set	Neutral data set	Adaptive data set
Predictor variables	MTWQ	Geo + AP	MTWQ	Geo + TS + MTWQ
Model deviance	7.79	4.56	8.36	3.58
Percentage explained	35.34	69.55	32.83	75.92
*p*‐value	0.109	0.000	0.023	0.000

Abbreviations: AP, annual precipitation (BIO12); Geo, geographic distance; MTWQ, mean temperature of the wettest quarter (BIO8); TS, temperature seasonality (BIO4).

**FIGURE 3:**
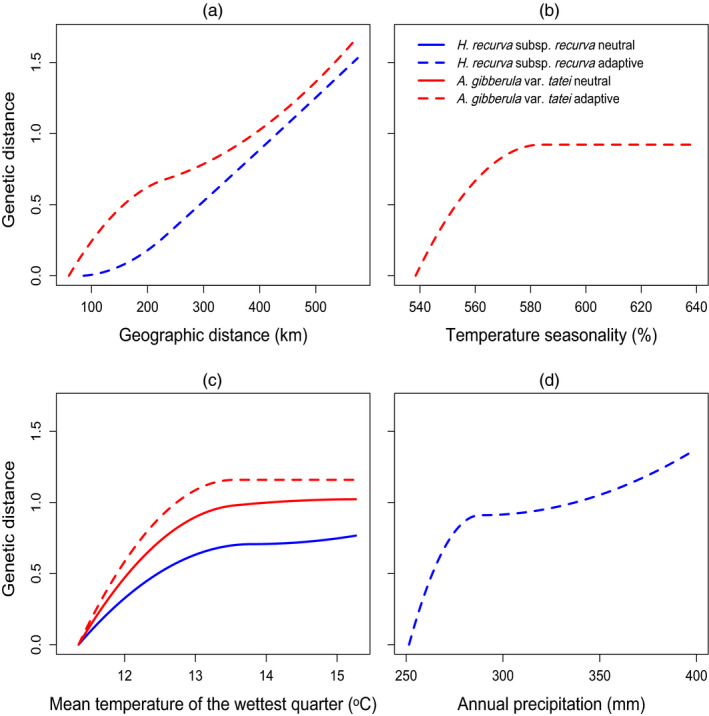
Generalized dissimilarity model‐fitted I‐splines showing allelic turnover across predictor variables for *Hakea recurva* subsp. *recurva* and *Amyema gibberula* var. *tatei*. Neutral data sets (14,848 and 1631 SNPs for each species, respectively) contained putatively neutral SNPs not identified as outliers, or with significant environment association, in any genome scan method. Adaptive data sets (35 and 36 SNPs for each species, respectively) contained putatively adaptive SNPs identified by two or more genome scan methods. Allelic turnover was only plotted if the data set had a significant relationship with the predictor variables: (a) geographic distance, (b) temperature seasonality (BIO4), (c) mean temperature of the wettest quarter (BIO8) and (d) annual precipitation (BIO12). Height of the curve indicates the total amount of allelic turnover associated with that predictor variable, when holding all other variables constant, and the shape indicates the rate of allelic turnover along the gradient

GDM analysis examined the relationship between genetic distance and environmental distance (i.e. geographic distance and climatic variables), with patterns varying by both predictor variable and species (Figure 3). Geographic distance showed a near linear relationship with genetic distance with the geographic spline predicting a gradual change in allelic turnover across the range (Table 3; Figure 3a). Additionally, geographic distance had the greatest spline height for both putatively adaptive data sets (1.54–1.67; Table 3), indicating that this was the most important predictor of allelic turnover. In contrast, all three climatic variables in the final GDM models had a nonlinear relationship with genetic variation (Figure 3b‐d). Temperature seasonality was a significant predictor of the putatively adaptive data set for the mistletoe, with the largest change in allelic turnover predicted to occur below 580% (Figure 3b). Mean temperature of the wettest quarter was the only predictor variable for the putatively neutral data sets in both species and was also an important predictor for the putatively adaptive data set in the mistletoe, with the largest change in allelic turnover predicted to occur below 13°C (Figure 3c). Finally, annual precipitation was a significant predictor of the putatively adaptive data set in the host, with the largest change in allelic turnover below 280 mm (Figure 3d).

**TABLE 3:** Relative importance of predictor variables in generalized dissimilarity models for *Hakea recurva* subsp. *recurva* and *Amyema gibberula* var. *tatei*. Putatively neutral data sets contained SNPs that were not identified as outliers, or with significant environment association, in any genome scan method (14,848 and 1631 SNPs for each species, respectively). Putatively adaptive data sets contained only SNPs identified by two or more genome scan methods (35 and 36 SNPs for each species, respectively). Relative importance values were obtained from the summations of the three spline coefficients for each significant predictor variables. Cells with no value indicate that the variable was not a significant predictor of that model

Relative importance	*Hakea recurva* subsp. *recurva*		*Amyema gibberula* var. *tatei*	
	Neutral data set	Adaptive data set	Neutral data set	Adaptive data set
Geo	‐	1.54	‐	1.67
IT (BIO3)	‐	‐	‐	‐
TS (BIO4)	‐	‐	‐	0.92
MTWQ (BIO8)	0.77	‐	1.02	1.16
MTDQ (BIO9)	‐	‐	‐	‐
AP (BIO12)	‐	1.36	‐	‐
PS (BIO15)	‐	‐	‐	‐
PWQ(BIO18)	‐	‐	‐	‐

Abbreviations: AP, annual precipitation; Geo, geographic distance; IT, isothermality; MTDQ, mean temperature of the driest quarter; MTWQ, mean temperature of the wettest quarter; PS, precipitation seasonality; PWQ, precipitation of the warmest quarter; TS, temperature seasonality.

**FIGURE 4:**
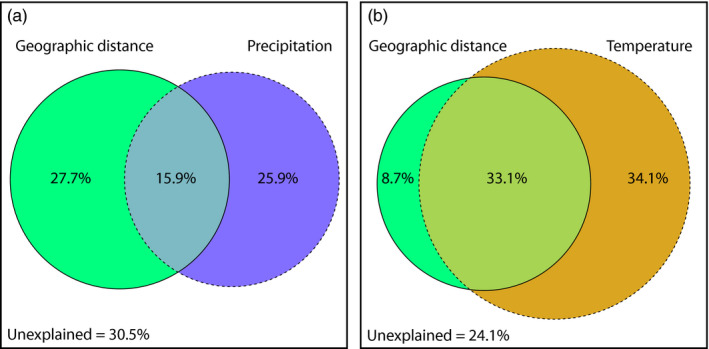
Partitioning of generalized dissimilarity model deviance by predictor variables for (a) *Hakea recurva* subsp. *recurva* and (b) *Amyema gibberula* var. *tatei*. Three sets of predictor variables were used (geographic distance, temperature and precipitation variables) for putatively adaptive data sets (35 and 36 SNPs for each species, respectively) that contained SNPs identified by two or more genome scan methods

Geographic distance explained approximately 43% of the GDM model deviance of putatively adaptive data sets for both species (Figure 4). Precipitation explained a similar proportion of GDM model deviance as geographic distance for the host, and no variation was explained by temperature for this species (Figure 4a). In contrast, precipitation did not explain any of the GDM model deviance for the mistletoe while temperature explained over 65%, although a large proportion of allelic turnover was jointly explained by geographic distance (Figure 4b). Unexplained variation in GDM model deviance was similar between species.

**FIGURE 5:**
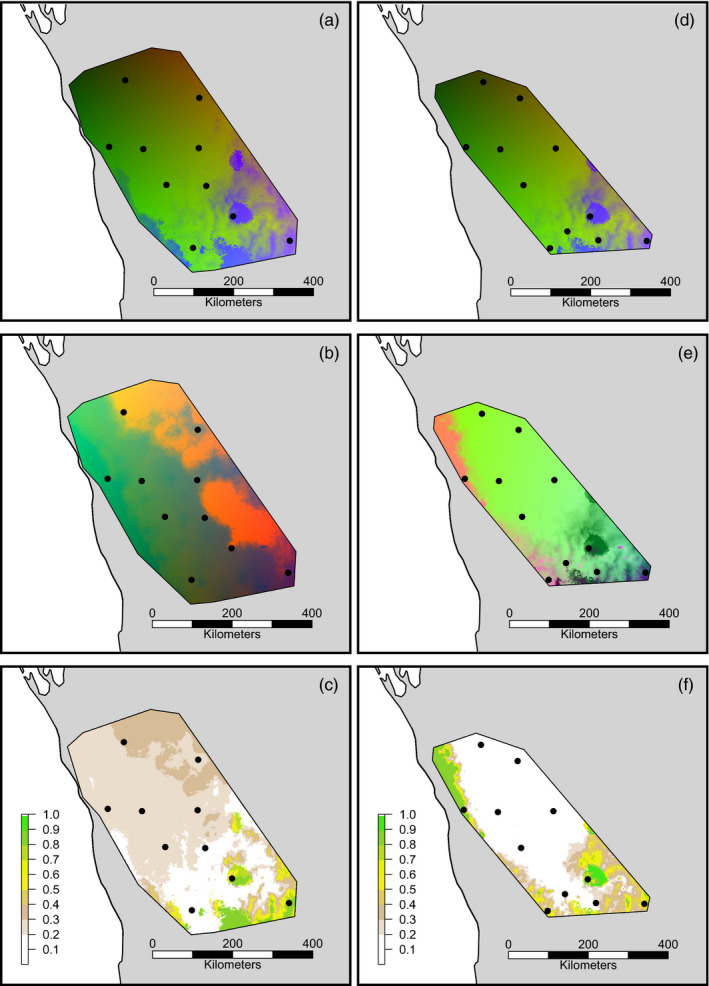
Spatial patterns of predicted genetic composition and differences in multivariate configuration (Procrustes residuals) for (a–c) *Hakea recurva* subsp. *recurva* and (d–f) *Amyema gibberula* var. *tatei*. Genetic compositions were derived using fitted generalized dissimilarity models to perform biologically informed transformations of significant climatic variables for putatively neutral data sets (a, d) and putatively adaptive data sets (b, e). Neutral data sets (14,848 and 1631 SNPs for each species, respectively) contained putatively neutral SNPs not identified as outliers, or with significant environment association, in any genome scan method. Adaptive data sets (35 and 36 SNPs for each species, respectively) contained putatively adaptive SNPs identified by two or more genome scan methods. Principal component analysis was used to reduce the transformed climatic variables into three principal components that were each assigned an RGB colour. The RGB maps do not have a scale bar but similarity of colours within each frame indicates similarity in predicted patterns of genetic composition. Differences in multivariate configuration between putatively neutral and putatively adaptive data sets were measured by Procrustes analysis (c, f). Procrustes residuals were scaled to allow direct comparisons between species

Spatial patterns of predicted genetic composition were similar for putatively neutral data sets of both species, but not for putatively adaptive data sets (Figure 5). Specifically, rapid turnover in genetic composition of putatively neutral data sets was similarly predicted in the south‐eastern region for both species (Figure 5a,d). In contrast, the turnover of genetic composition for putatively adaptive data sets was predicted to occur more rapidly in the eastern region for the host (Figure 5b) but the western and southern regions for the mistletoe (Figure 5e). Procrustes residuals, which compared multivariate configuration between putatively neutral and putatively adaptive data sets, varied spatially across the distribution of both species. In general, residuals were higher in the southern half of the range for each species, indicating less congruence between SNP data sets (Figure 5c,f).

## 4 DISCUSSION

Our investigation of genome‐wide variation along climatic gradients in a specialist mistletoe and its host found that putatively adaptive genetic variation was associated with different climatic variables for each species. These differences between host and parasite in the turnover of putatively adaptive SNPs contrasted with the similarity in turnover of putatively neutral SNPs and support our alternative hypothesis that these species respond differently to climatic variables. Specifically, temperature was relatively more important in predicting allelic turnover for the mistletoe, while precipitation was more important for the host. This could reflect a parasitic life history as parasitic plants have different water acquisition strategies and transpiration rates to their hosts. While genome‐wide markers have been used to examine climate adaptation in numerous taxa, applying these methods to a comparative study of nonmodel organisms in a host–parasite system has presented some distinct challenges, which we discuss below.

### 4.1 Associations with climatic variables in a mistletoe‐host system

Mistletoes and their hosts have been previously recorded as having similar relationships between physiological parameters and climate (Scalon & Wright, 2015). However, this is the first study to compare the associations of genome‐wide variation along climatic gradients in mistletoe–host systems, which deepens our understanding of adaptation and provides insight into the evolutionary processes that underlie previously observed phenomena. Despite similar associations of putatively neutral genetic variation between the mistletoe and its host, associations of putatively adaptive variation with climatic variables were different. Specifically, GDM analysis indicated putatively adaptive variation in the mistletoe to be associated with temperature. Seasonality and mean temperature of the wettest quarter were particularly associated with the mistletoe, as also correlated with population differentiation in another mistletoe (Ramírez‐Barahona et al., 2017). One explanation for the importance of temperature is that mistletoes (and other parasitic plants) must maintain a hydrostatic gradient to draw water from hosts, which is achieved through increased transpiration rates relative to host plants (Ehleringer & Marshall, 1995; Stewart & Press, 1990). As temperature influences transpiration rates, temperature may provide a greater selective pressure for the mistletoe in comparison to the host. While these patterns of genome‐wide variation are consistent with local adaptation, further validation of the role of temperature is needed. Experimental work in other host–parasite systems have found temperature to influence local adaptation (Laine, 2007, 2008) and the performance of mistletoe populations (Sangüesa‐Barreda et al., 2018).

GDM analysis showed putatively adaptive variation in the host to be associated with precipitation, which is consistent with findings of other studies on autotrophic plants (e.g. Manel et al., 2012; Shryock et al., 2017; Steane et al., 2017; Walters et al., 2020). However, precipitation was not found to associate with putatively adaptive variation in the mistletoe. This was despite recent experimental evidence suggesting that population differentiation in mistletoes can vary along gradients of water availability and precipitation seasonality (Ramírez‐Barahona et al., 2017). One explanation for the difference in relative importance of precipitation in this study could be the different water acquisition strategies between the mistletoe and its host, which may respond differently to abiotic selective pressures. Specifically, acquisition of water from host plants (rather than the abiotic environment) may provide a buffer between the mistletoe and climatic conditions. This could provide a more uniform environment in terms of water availability and, therefore, reduced selection pressures due to seasonal variability in precipitation.

Similar observations on associations of genome‐wide variation with climatic variables have also been found for a generalist parasite and sympatric autotroph (Walters et al., 2020), although association with climatic variables does not necessarily imply that these variables directly shape adaptive genetic variation. For instance, the variables assessed here may correlate with other environmental gradients (e.g. solar radiation, altitude; Garnier‐Géré & Ades, 2001; Gauli et al., 2015) that may be true drivers of adaptation in these species. Covariation between climatic variables and geographic distance can also make it difficult to determine which variable shapes adaptive genetic variation, or whether patterns are jointly influenced by multiple variables. Despite this study providing evidence consistent with local adaptation, additional sampling and experimental work is needed to explicitly confirm the effect of climate adaptation in these species.

In this study, we found putatively adaptive variation to positively correlate with geographic distance, similar to that in other plant species (e.g. Shryock et al., 2017). This could be the result of correlation between clines in abiotic variables that drive adaptation and geographic distance across the study area. Another explanation could be that mistletoes may adapt to populations of specific hosts and specialist seed dispersal vectors within a specific geographic area. Indeed, geographic distance has been previously found to influence neutral genetic structure between populations of mistletoes (Nyagumbo et al., 2017; Yule et al., 2016) and other parasitic species (Feurtey et al., 2016), although not always for specialist mistletoes (Jerome & Ford, 2002). For both species in our study, the most south‐eastern population was notably different from other populations, which is likely due to the isolated occurrence of this population on the edge of the species’ range.

### 4.2 Factors affecting the study of selection pressure in nonmodel organisms using genome‐wide markers

Genome‐wide markers have been used to identify signals of selection across many taxonomic groups (reviewed in Ahrens et al., 2018); yet applying these methods to a comparative study in a host–parasite system has posed some distinct challenges. Firstly, demographic and life histories can differ widely between species; for instance, parasites generally have much shorter generation times (Huyse et al., 2005). Faster generation times lead to more frequent genome replication that collects more DNA mutations per unit of time, and therefore, adaptation can proceed more rapidly (Bromham et al., 2013; Smith & Donoghue, 2008). Mutation rates can also vary between parasites (Nieberding & Olivieri, 2007); therefore, the detection of genome‐wide variation in other host–parasite systems may differ to that observed here. Population sizes were also observed to differ between our species, which would influence the genetic variability within populations (Charlesworth, 2009).

Another complicating factor for our study is that both nonmodel species lack reference genomes or transcriptomes, and consequently, we have not been able to verify the gene function of putatively adaptive SNPs. While signals of selection can still be obtained for species that lack prior genomic knowledge (Savolainen et al., 2013), it is likely that most SNPs identified as outliers, or those with significant environment association, are not under direct selection, but rather are physically linked to loci under selection (i.e. genetic hitchhiking; reviewed by Barton, 2000). The exact effect of genetic hitchhiking on genome scans depends upon a number of evolutionary parameters (Lotterhos & Whitlock, 2015), the majority of which are unknown for our study species. Future work could expand upon these findings by using reference genomes to map the gene function of both SNPs identified as outliers and those with significant environment association (Bragg et al., 2015; Breed et al., 2019; Tiffin & Ross‐Ibarra, 2014).

Lastly, while our study has enabled association of putatively adaptive variation with different climatic gradients, it does not provide insight into the genetic architecture of climate adaptation in these species *per se*. For instance, polygenic adaptation of many loci with small effect that result in phenotypic changes may be difficult to detect with genome scans in comparison to loci with a single, large effect (Le Corre & Kremer, 2012; Pritchard & Di Rienzo, 2010). This could influence the number of SNPs identified as putatively adaptive as the ratios of loci with small and large effects may differ between these species, although this information was not available *a priori*. Furthermore, we also found stronger population differentiation in the mistletoe and this is known to influence the detection of loci under selection (Flanagan et al., 2018; de Villemereuil et al., 2014). High population differentiation in mistletoes could be due to their complex requirements for specialist seed dispersal vectors, specific hosts and the availability of abiotic niches (e.g. Lira‐Noriega & Peterson, 2014; Ramírez‐Barahona et al., 2017) that may ultimately limit the dispersal of mistletoes across the landscape. Although population differentiation was accounted for in our EA analyses, combining these results with phenotypic data could further our understanding of local climatic adaptation between mistletoes and their hosts.

### 4.3 Comparison with other host–parasite systems

Our findings on the association of putatively adaptive variation with climatic variables in a mistletoe–host system were similar to those of a previous study on a generalist parasite and co‐occurring autotroph (Walters et al., 2020). For both parasitic plants, there was a stronger association of putatively adaptive variation to temperature variables, in comparison to precipitation variables for host species. While these results could be indicative of the parasitic life history and suggest that adaptive responses may vary between species with different water acquisition strategies, further examination is needed to confirm this pattern in other parasitic plants. Furthermore, patterns on the association of putatively adaptive variation with climatic variables may differ in other host–parasite systems that often have fewer differences in gene flow and population structure between parasites and hosts (e.g. Dybdahl & Lively, 1996; Feurtey et al., 2016; McCoy et al., 2005). Examining the association of genome‐wide variation could be crucial to understanding climate adaptation, particularly as parasite evolution depends upon the physical environment (Laine, 2008). Therefore, extending this work to other host–parasite systems would further increase our understanding of the association of putatively adaptive variation to climatic variables in natural populations.

## CONFLICT OF INTEREST

None declared.

## AUTHOR CONTRIBUTIONS

All authors conceptualized the study design. S.J.W. collected field samples, extracted the DNA and analysed the data with input from co‐authors. S.J.W., P.N., T.P.R. and M.B. interpreted the results. S.J.W. wrote the manuscript. All authors read, edited and approved the final manuscript.

## Supporting information

Supplementary Material

## Data Availability

The data that support the findings of this study are openly available in the DRYAD Digital Repository at http://doi.org/10.5061/dryad.k3j9kd56n
