# Supplementary material for: Association of putatively adaptive genetic variation with climatic variables differs between a parasite and its host
Source: Evol Appl. 2021 Apr 8;14(7):1732–46. doi: 10.1111/eva.13234 (PMC8288004; doi:10.1111/eva.13234)
Supplement: Supplementary file 1 — Supplementary Material [file EVA-14-1732-s001.docx]

**Supplementary material**

for

**Association of putatively adaptive genetic variation with climatic variables differs between a parasite and its host**

**Table of Contents:**

| **Table S1.** Coordinates and climatic data for *Hakea recurva* subsp. *recurva* populations sampled across south-western Australia. | **Page 2** |
| --- | --- |
| **Table S2.** Coordinates and climatic data for *Amyema gibberula* var. *tatei* populations sampled across south-western Australia. | **Page 3** |
| **Figure S3.** Climate maps of the seven uncorrelated climatic variables in south-western Australia. | **Page 4** |
| **Figure S2.** Screeplots for principal component analysis of (A) *Hakea recurva* subsp. *recurva* and (B) *Amyema gibberula* var. *tatei*. | **Page 5** |

**Table S1**. Coordinates and climatic data for *Hakea recurva* subsp. *recurva* populations sampled across south-western Australia. Latitude and longitude are presented in the Geocentric Datum of Australia (GDA94) coordinate system. Mean ± SD are provided for seven least-correlated bioclimatic variables used in environmental association analysis and generalised dissimilarity modelling (IT: isothermality, BIO3; TS: temperature seasonality, BIO4; MTWQ: mean temperature of the wettest quarter, BIO8; MTDQ: mean temperature of the driest quarter, BIO9; AP: annual precipitation, BIO12; PS: precipitation seasonality, BIO15; PWQ: precipitation of the warmest quarter, BIO18).

| **Code** | **Population** | **Latitude** | **Longitude** | **No.** | **IT**  **(%)** | **TS**  **(ᵒC)** | **MTWQ**  **(ᵒC)** | **MTDQ**  **(ᵒC)** | **AP**  **(mm)** | **PS**  **(%)** | **PWQ**  **(mm)** |
| --- | --- | --- | --- | --- | --- | --- | --- | --- | --- | --- | --- |
| BOO | Boogardie | -28.039939 | 117.674462 | 16 | 42.8 ± 0.14 | 653 ± 1.24 | 14.2 ± 0.01 | 20.3 ± 0.03 | 266 ± 0.87 | 40.0 ± 0.44 | 68.1 ± 0.25 |
| DEP | Depot Hill | -29.143822 | 115.346400 | 16 | 49.7 ± 0.00 | 538 ± 0.00 | 15.3 ± 0.00 | 24.6 ± 0.00 | 397 ± 0.00 | 76.2 ± 0.00 | 39.0 ± 0.00 |
| JIB | Jibberding | -30.000985 | 116.826976 | 16 | 45.8 ± 0.01 | 586 ± 0.02 | 14.0 ± 0.00 | 25.2 ± 0.01 | 298 ± 0.34 | 53.0 ± 0.09 | 36.0 ± 0.00 |
| KAR | Karroun Hill | -30.024129 | 117.856322 | 16 | 44.4 ± 0.00 | 613 ± 0.48 | 13.1 ± 0.01 | 22.2 ± 0.02 | 284 ± 0.34 | 46.4 ± 0.12 | 45.0 ± 0.00 |
| KOO | Koolanooka | -29.193801 | 116.228194 | 16 | 45.0 ± 0.00 | 588 ± 0.00 | 14.3 ± 0.00 | 22.3 ± 0.00 | 320 ± 0.00 | 55.3 ± 0.00 | 46.0 ± 0.00 |
| KOR | Koora | -31.260612 | 120.011578 | 16 | 45.6 ± 0.00 | 544 ± 0.00 | 11.3 ± 0.00 | 22.2 ± 0.00 | 292 ± 0.00 | 43.6 ± 0.00 | 47.0 ± 0.00 |
| NIN | Ninghan | -29.172953 | 117.660768 | 16 | 43.8 ± 0.14 | 628 ± 1.83 | 13.6 ± 0.06 | 20.4 ± 2.81 | 284 ± 1.15 | 46.4 ± 0.62 | 52.5 ± 0.52 |
| WOO | Woolgorong | -27.644765 | 115.761262 | 16 | 46.0 ± 0.07 | 615 ± 0.79 | 15.2 ± 0.00 | 23.9 ± 0.04 | 252 ± 1.26 | 52.6 ± 0.21 | 45.6 ± 0.50 |
| YAN | Yanneymooning | -30.708080 | 118.547847 | 16 | 45.0 ± 0.03 | 584 ± 0.55 | 11.6 ± 0.02 | 24.3 ± 0.04 | 267 ± 0.89 | 47.1 ± 0.17 | 37.3 ± 0.45 |
| YOR | Yorkrakine Rock | -31.421116 | 117.512880 | 16 | 45.5 ± 0.02 | 548 ± 0.26 | 12.9 ± 0.01 | 23.2 ± 0.02 | 320 ± 0.50 | 51.2 ± 0.17 | 40.0 ± 0.00 |
| **TOTAL** | | | | 160 | 45.4 ± 1.70 | 590 ± 36.5 | 13.6 ± 1.30 | 22.9 ± 1.80 | 298 ± 39.3 | 51.2 ± 9.50 | 45.6 ± 8.90 |

**Table S2**. Coordinates and climatic data for *Amyema gibberula* var. *tatei* populations sampled across south-western Australia. Latitude and longitude are presented in the Geocentric Datum of Australia (GDA94) coordinate system. Mean ± SD are provided for seven least-correlated bioclimatic variables used in environmental association analysis and generalised dissimilarity modelling (IT: isothermality, BIO3; TS: temperature seasonality, BIO4; MTWQ: mean temperature of the wettest quarter, BIO8; MTDQ: mean temperature of the driest quarter, BIO9; AP: annual precipitation, BIO12; PS: precipitation seasonality, BIO15; PWQ: precipitation of the warmest quarter, BIO18).

| **Code** | **Population** | **Latitude** | **Longitude** | **No.** | **IT**  **(%)** | **TS**  **(ᵒC)** | **MTWQ**  **(ᵒC)** | **MTDQ**  **(ᵒC)** | **AP**  **(mm)** | **PS**  **(%)** | **PWQ**  **(mm)** |
| --- | --- | --- | --- | --- | --- | --- | --- | --- | --- | --- | --- |
| BIL | Billyacatting Hill | -31.042439 | 117.959293 | 16 | 45.4 ± 0.01 | 555 ± 0.10 | 11.8 ± 0.00 | 25.3 ± 0.00 | 320 ± 0.25 | 44.5 ± 0.12 | 44.1 ± 0.25 |
| DEP | Depot Hill | -29.143822 | 115.346400 | 16 | 49.7 ± 0.00 | 538 ± 0.00 | 15.3 ± 0.00 | 24.6 ± 0.00 | 397 ± 0.00 | 76.2 ± 0.00 | 39.0 ± 0.00 |
| GAB | Gabyon | -28.043533 | 116.736323 | 10 | 44.6 ± 0.06 | 639 ± 0.28 | 14.3 ± 0.00 | 23.7 ± 0.00 | 249 ± 1.03 | 49.3 ± 0.14 | 57.0 ± 0.00 |
| JIB | Jibberding | -30.000985 | 116.826976 | 16 | 45.8 ± 0.01 | 586 ± 0.02 | 14.0 ± 0.00 | 25.2 ± 0.01 | 298 ± 0.34 | 53.0 ± 0.09 | 36.0 ± 0.00 |
| KOO | Koolanooka | -29.193801 | 116.228194 | 16 | 45.0 ± 0.00 | 588 ± 0.00 | 14.3 ± 0.00 | 22.3 ± 0.00 | 320 ± 0.00 | 55.3 ± 0.00 | 46.0 ± 0.00 |
| KOR | Koora | -31.260612 | 120.011578 | 16 | 45.6 ± 0.00 | 544 ± 0.00 | 11.3 ± 0.00 | 22.2 ± 0.00 | 292 ± 0.00 | 43.6 ± 0.00 | 47.0 ± 0.00 |
| NIN | Ninghan | -29.172953 | 117.660768 | 12 | 43.7 ± 0.11 | 629 ± 1.93 | 13.5 ± 0.07 | 19.7 ± 2.01 | 285 ± 0.90 | 46.3 ± 0.58 | 52.7 ± 0.49 |
| SAN | Sandford Rocks | -31.242659 | 118.760163 | 16 | 45.4 ± 0.00 | 558 ± 0.00 | 12.3 ± 0.00 | 23.1 ± 0.00 | 335 ± 0.00 | 40.5 ± 0.00 | 53.0 ± 0.00 |
| WOO | Woolgorong | -27.678167 | 115.789340 | 12 | 46.0 ± 0.09 | 615 ± 1.38 | 15.2 ± 0.11 | 23.8 ± 0.14 | 253 ± 2.84 | 52.6 ± 0.22 | 45.5 ± 0.52 |
| YAN | Yanneymooning | -30.708080 | 118.547847 | 12 | 45.0 ± 0.03 | 584 ± 0.60 | 11.6 ± 0.02 | 24.3 ± 0.04 | 266 ± 0.79 | 47.2 ± 0.15 | 37.2 ± 0.39 |
| YOR | Yorkrakine Rock | -31.421116 | 117.512880 | 12 | 45.5 ± 0.03 | 548 ± 0.30 | 12.9 ± 0.01 | 23.2 ± 0.03 | 320 ± 0.58 | 51.1 ± 0.19 | 40.0 ± 0.00 |
| **TOTAL** | | | |  | 45.7 ± 1.46 | 577 ± 32.0 | 13.3 ± 1.37 | 23.5 ± 1.63 | 308 ± 40.4 | 51.1 ± 9.69 | 44.9 ± 6.36 |

**Supplementary Figure S1**. Climate maps of the seven uncorrelated climatic variables in south-western Australia.


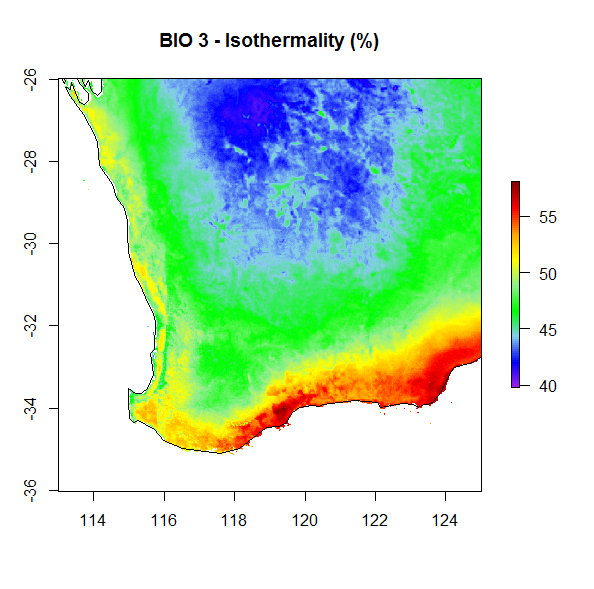

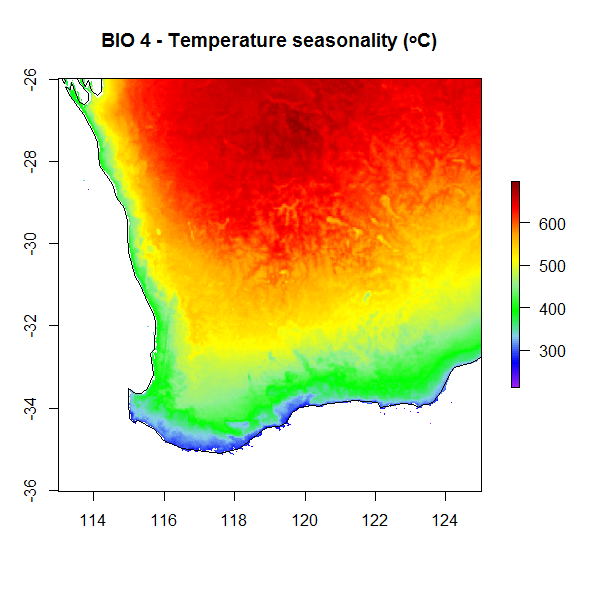

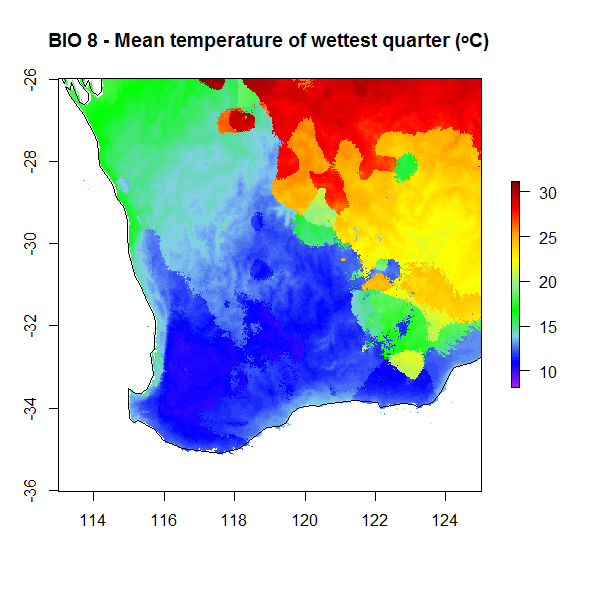

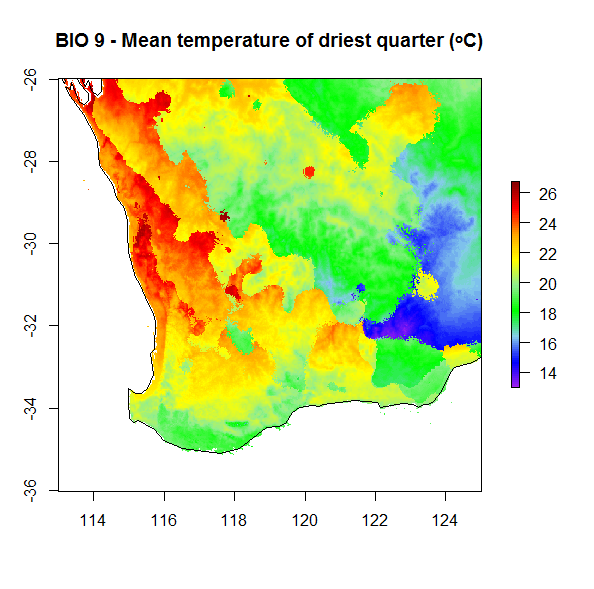

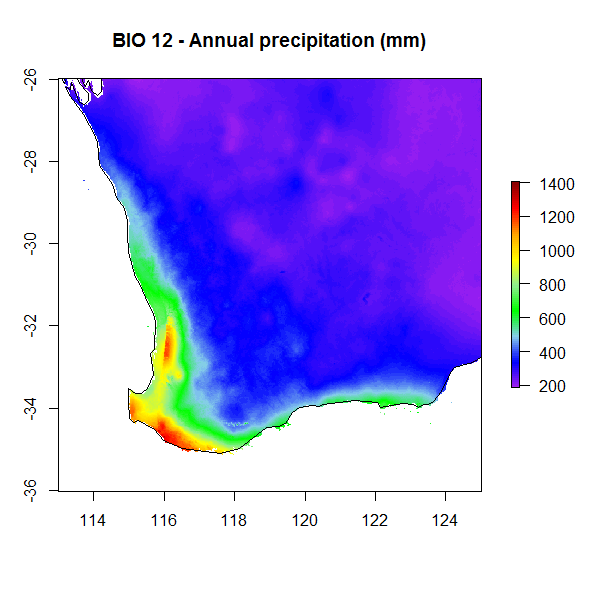

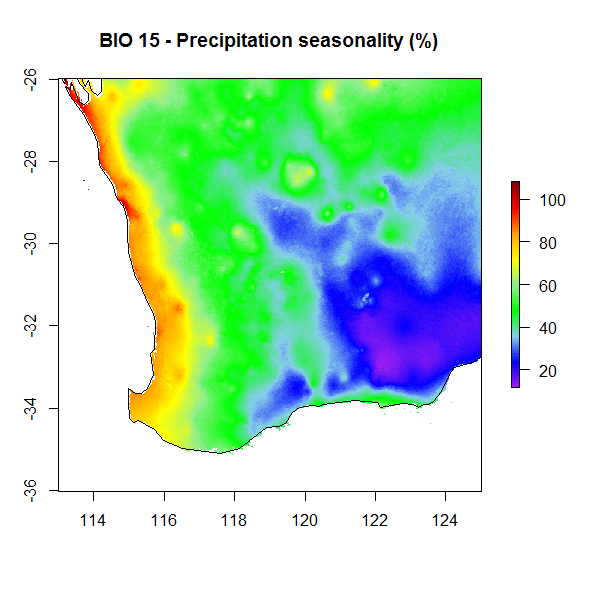

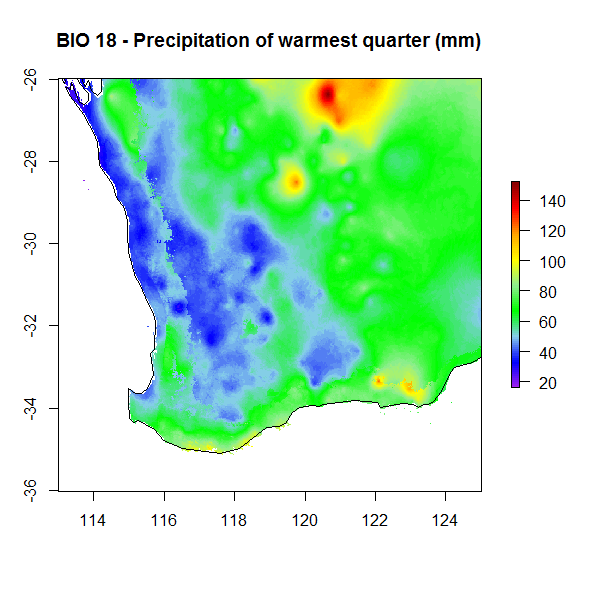


**Supplementary Figure S2.** Screeplots for principal component analysis of (A) *Hakea recurva* subsp. *recurva* and (B) *Amyema gibberula* var. *tatei*.


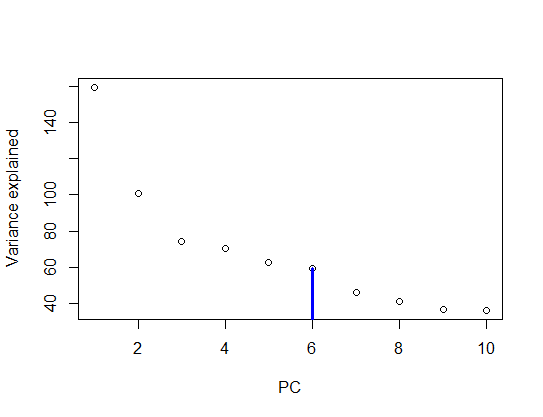


**(B)**


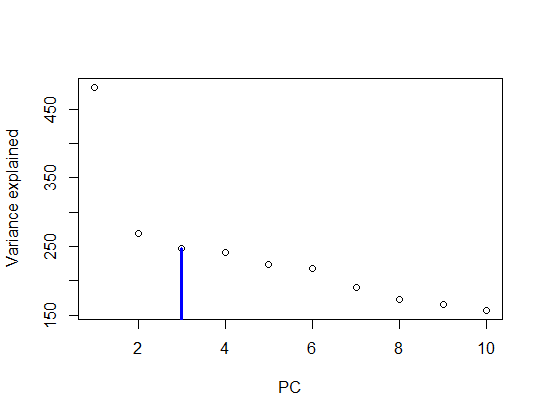


**(A)**
